# Drop Jump Asymmetry is Associated with Reduced Sprint and Change-of-Direction Speed Performance in Adult Female Soccer Players

**DOI:** 10.3390/sports7010029

**Published:** 2019-01-21

**Authors:** Chris Bishop, Anthony Turner, Sean Maloney, Jason Lake, Irineu Loturco, Tom Bromley, Paul Read

**Affiliations:** 1 Faculty of Science and Technology, London Sports Institute, Middlesex University, London NW4 1RL, UK; A.N.Turner@mdx.ac.uk; 2 Department of Sport Science and Physical Activity, University of Bedfordshire, Bedford MK41 9EA, UK; Sean.Maloney@beds.ac.uk; 3 Department of Sport and Exercise Sciences, University of Chichester, West Sussex PO19 6PE, UK; J.Lake@chi.ac.uk; 4 Department of Sport Science and Research, Nucleus of High Performance in Sport, Sao Paulo 04753060, Brazil; irineu.loturco@terra.com.br; 5 Milton Keynes Dons Football Club, Stadium MK, Grafton Street, Milton Keynes MK1 1ST, UK; Tom.Bromley@mkdons.com; 6 Athlete Health and Performance Research Centre, Aspetar Orthopaedic and Sports Medicine Hospital, Doha PO Box 29222, Qatar; Paul.Read@aspetar.com

**Keywords:** inter-limb differences, jumping, performance reduction

## Abstract

Studies that examine the effects of inter-limb asymmetry on measures of physical performance are scarce, especially in adult female populations. The aim of the present study was to establish the relationship between inter-limb asymmetry and speed and change-of-direction speed (CODS) in adult female soccer players. Sixteen adult players performed a preseason test battery consisting of unilateral countermovement jump (CMJ), unilateral drop jump (DJ), 10 m, 30 m, and 505 CODS tests. Inter-limb asymmetry was calculated using a standard percentage difference equation for jump and CODS tests, and Pearson’s *r* correlations were used to establish a relationship between asymmetry and physical performance as well as asymmetry scores themselves across tests. Jump-height asymmetry from the CMJ (8.65%) and DJ (9.16%) tests were significantly greater (*p* < 0.05) than asymmetry during the 505 test (2.39%). CMJ-height asymmetry showed no association with speed or CODS. However, DJ asymmetries were significantly associated with slower 10 m (*r* = 0.52; *p* < 0.05), 30 m (*r* = 0.58; *p* < 0.05), and 505 (*r* = 0.52–0.66; *p* < 0.05) performance. No significant relationships were present between asymmetry scores across tests. These findings suggest that the DJ is a useful test for detecting existent between-limb asymmetry that might in turn be detrimental to speed and CODS performance. Furthermore, the lack of relationships present between different asymmetry scores indicates the individual nature of asymmetry and precludes the use of a single test for the assessment of inter-limb differences.

## 1. Introduction

Inter-limb asymmetry has been a common line of investigation in recent years, with numerous studies reporting the prevalence of side-to-side differences during various tests of physical capacity [1,2,3]. When investigating inter-limb asymmetry, numerous factors should be considered when choosing the appropriate test for athletes, such as test reliability, needs of the athlete and availability of equipment [4]. Whilst these factors must be given careful consideration when measuring asymmetry, it is difficult to argue against the importance of strength and power in almost all athletes. Measuring asymmetry during strength tasks has been conducted using the back squat [5], isokinetic dynamometry [6,7,8], and isometric tasks such as the mid-thigh pull or isometric squat [9,10,11]. In addition, strength asymmetry (typically peak force or torque) have been shown to be negatively correlated with jump performance [9], speed and change-of-direction speed (CODS) [8], and kicking accuracy [11]. Whilst useful, such test methods often require substantial time commitments and expensive equipment, making them harder to conduct for practitioners in the field. Therefore, alternative methods of assessing inter-limb asymmetry may be required.

Jump testing is often included as part of routine fitness testing and offers practitioners a viable and time-efficient method of assessment that often reflects the movement patterns accompanied in sport [4,12,13]. Furthermore, previous literature has shown that athletes who exhibit inter-limb differences (from unilateral jump tests) >10%, are four times more likely to re-rupture their anterior cruciate ligament [14]. Previous studies have used bilateral and unilateral procedures to assess asymmetry inclusive of: The countermovement jump (CMJ) [15,16,17], standing broad jump (SBJ) [15,17,18], and drop jump (DJ) [2,19], with multiple metrics available to be assessed such as jump height, distance, and reactive strength index (RSI). When using these tests to examine the effects that inter-limb asymmetry has on measures of physical performance, the results are equivocal [20,21,22]. Maloney et al. [2] showed that jump-height asymmetry during the unilateral drop jump test were associated with a slower change-of-direction speed (CODS) performance (*r* = 0.6) in healthy male adults. Furthermore, faster participants demonstrated significantly smaller jump-height asymmetry (2.4%) than slower participants (7.2%). Similarly, Bishop et al. [16] showed that jump-height asymmetry of 12.5% from the unilateral CMJ was associated with slower linear speed and jump performance in academy youth female soccer players. In contrast, Lockie et al. [17] reported jump-height asymmetry of 10.4% from the unilateral CMJ with no effect on speed or CODS performance in a male collegiate sample. Further to this, Dos’Santos et al. [18] reported jump-distance asymmetry of 6.3% from a unilateral SBJ test, but showed no association with two CODS tests that were also assessed in a male collegiate population. Given the conflicting findings in the literature, further research is warranted to establish the relationship between vertical jumping asymmetry and measures of physical performance. Furthermore, and to the authors’ knowledge, such studies in an adult female athlete population do not exist to date.

Therefore, the aim of the present study was to establish the relationship between inter-limb asymmetry from unilateral jump tests and both linear speed and CODS performance in an adult female soccer sample. Given the scarcity of data assessing the effects of asymmetry in female populations, a true hypothesis was difficult to generate; however, it was hypothesized that larger between-limb asymmetry would be associated with reduced physical performance [2,16].

## 2. Materials and Methods

### 2.1. Study Design

The present study undertook a preseason fitness testing battery with adult female soccer athletes from a Category 3 Regional Talent Centre at a professional club. The test battery consisted of a unilateral CMJ, unilateral DJ, 10 m and 30 m sprint, and 505 CODS test. Owing to the previously reported importance of jumping, sprinting, and CODS for soccer players [23,24,25,26], these tests were deemed appropriate for the population. Given the volume of measurements being conducted, the test battery was split over two training sessions to minimize the risk of fatigue impacting scores. Test sessions were performed at the same time of day in the evening from 19:00–21:00. Both jump tests were conducted together at the start of a training session and the sprint and CODS tests were conducted at the start of a separate training session 48 h later.

### 2.2. Participants

Sixteen adult female soccer players (age: 20.56 ± 1.26 years; height: 1.65 ± 0.06 m; body mass: 63.85 ± 7.02 kg) participated in this study. All participants had at least six years of competitive soccer experience and had taken part in structured strength and conditioning training twice per week with a minimum of 12 months resistance and ballistic training experience. All participants were free from injury at the time of testing and had been for a minimum of three months, noting that testing occurred at the start of the preseason. Informed consent was provided in line with the soccer club’s policy, and ethical approval was granted from the London Sport Institute research and ethics committee.

### 2.3. Procedures

Test sessions began with a dynamic warmup specific to the intended test outcomes. Specifically, a series of dynamic stretches were completed, targeting first the lower body and thoracic spine, and including multi-planar lunges, inchworms, and spidermans. Following this, the first test session required each player to perform three practice jumps on each limb at 60, 80, and 100% of perceived maximal effort for both tests. For the second test session, three practice trials of a 30 m sprint and a 505 CODS test were performed at the same perceived intensities. These sub-maximal trials were conducted to act as test familiarization in the field. Three minutes of passive rest was provided before the start of data collection.

Unilateral Countermovement Jump. This test was performed using an optical measurement system OptoJump (Microgate, Bolzano, Italy), which has reported near perfect reliability and been shown to be strongly correlated with force platforms for the assessment of flight time [27]. All jumps were performed on a hard wooden surface and initiated by executing a countermovement to a self-selected depth before accelerating vertically as quickly as possible into the air. The jumping leg was required to remain fully extended during the flight phase, with hands fixed to hips throughout the jump before landing back in between the optical measurement system. The non-jumping leg started slightly flexed at the hip and knee joints, so that the foot would hover approximately parallel with the medial malleolus of the jumping leg. The recorded metric for all CMJ tests was jump height (calculated from the flight time method). Two jumps were performed on each leg, separated by a 30-second rest period, with the highest jump subsequently used for data analysis.

Unilateral Drop Jump. The DJ was also performed using the OptoJump measurement system with a box height of 18 cm chosen in line with previous research using this test [2,19]. With hands fixed on hips, participants were required to step off the box with their designated test leg, which subsequently landed on a hard wooden surface between the optical measurement system below. Upon landing, participants were instructed to minimize ground contact time and jump as high as possible thereafter in line with previous suggestions [2,19]. Two jumps were performed on each leg, separated by a 30-second rest period. Recorded metrics included jump (height calculated from the flight time method), ground contact time (time spent in contact with the ground after the initial landing but prior to take-off), and RSI (calculated using the equation flight time/ground contact time [2]). The trial with the greatest RSI was subsequently used for further analysis.

10 and 30 m Sprints. Electronic timing gates (Brower Timing Systems, Draper, UT, USA) were positioned at 0, 10, and 30 m, enabling multiple splits to be measured during a single sprint. Players started the test in a staggered two-point stance with toes positioned 30 cm behind the start line, so as not to break the beam of the timing gates prior to the initiation of the test. When ready, subjects sprinted through the final set of timing gates, allowing 10 and 30 m split times to be recorded to the nearest hundredth of a second. Two trials were performed on a 4G synthetic surface and separated by a 90-second rest period, with the fastest trial used for further analysis.

505 Change-of-Direction Speed Test. Fifteen meters were measured out and electronic timing gates were positioned at the 10 m mark, while the 15 m point was positioned on the goal line to ensure that players had an obvious target as they approached the turning point. Players sprinted 15 m on the 4G synthetic and then performed a 180° turn off both the right and left legs, with a total of two trials completed for each leg. The time started when players broke the electronic beam at the 10 m mark and after turning 180°, before sprinting back through the timing gates to complete a recorded distance of 10 m. A 90-second rest period was provided between trials with the fastest trial used for subsequent data analysis.

### 2.4. Statistical Analysis

Test scores were initially recorded as means and standard deviations (SD) in Microsoft Excel before being transferred to SPSS (version 24.0; SPSS, Inc., Armonk, NY, USA). All data were checked for normality using the Shapiro–Wilk test, and within-session reliability of test measures were computed using a two-way random intraclass correlation coefficient (ICC) with absolute agreement inclusive of 95% confidence intervals (relative reliability) and the coefficient of variation (CV) (absolute reliability). Interpretation of ICC values was in accordance with previous research by Koo and Li [28], where values >0.9 = excellent, 0.75–0.9 = good, 0.5–0.75 = moderate, and <0.5 = poor. CV values were considered acceptable if <10% [29].

Mean inter-limb asymmetry values (CMJ = jump height; DJ = jump height; ground contact time; RSI; 505 = total time) were computed using the same standard percentage difference equation: 100/(maximum value from left and right)*(minimum value from left and right)*−1 + 100, which has been suggested as accurate for the calculation of asymmetries from unilateral tests [16,30]. To compute the direction of asymmetry, an “IF function” was added to the end of the asymmetry formula: *IF(left < right, 1,−1). Thus, if an asymmetry score was positive, the larger value was reported on the right limb, and vice versa for a negative asymmetry score [31]. Pearson’s *r* correlations were conducted to establish the relationship between inter-limb asymmetry scores and fitness test scores, as well as between asymmetry scores across tests, with statistical significance set at *p* < 0.05. A one-way repeated measures ANOVA was conducted to determine the systematic bias between mean asymmetry values, with statistical significance set at *p* < 0.05.

## 3. Results

All data were normally distributed (*p* > 0.05) and within-session reliability data can be viewed in Table 1. All tests reported good to excellent reliability (ICC = 0.80–0.94) and acceptable variability (CV ≤ 7.51%). When comparing group mean asymmetry scores, jump-height asymmetry (in both tests) was significantly greater than CODS asymmetry (Table 1).

Pearson’s *r* correlations between inter-limb asymmetry scores and fitness tests are shown in Table 2. No significant relationships were found between unilateral CMJ asymmetry and either speed or CODS performance. In contrast, jump-height and RSI asymmetry during the DJ were significantly correlated with 10 m (*r* = 0.52), 30 m (*r* = 0.58), and 505 (*r* = 0.52 to 0.66) performances, indicating that larger asymmetries are indicative of slower sprint and CODS times. Pearson’s *r* correlations between inter-limb asymmetry scores across tests are shown in Table 3; no significant relationships were present between tests.

**Figure 1:**
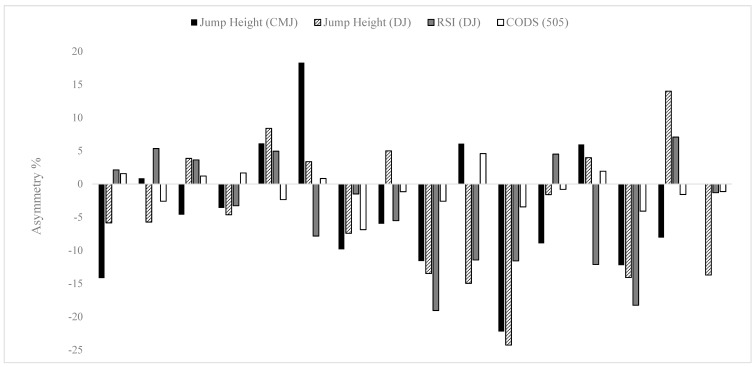
Individual asymmetry values (*n* = 16) for jump height during the unilateral countermovement jump (CMJ), jump height and reactive strength index (RSI) during the unilateral drop jump (DJ), and 505 change-of-direction speed (CODS) tests. N.B: Above the 0 line = larger score achieved on the right leg; below the 0 line = larger score achieved on the left leg.

Individual asymmetry values for each player are presented in Figure 1 for jump height (CMJ), jump height and RSI (DJ), and total time (505). Individual asymmetry values ranged from 0.0%–22.2% in the CMJ, 1.3%–24.3% in the DJ, and 0.8%–6.9% in the 505 test, highlighting the vast range of between-limb deficits in adult female soccer players. 

## 4. Discussion

The aim of the present study was to establish the relationship between inter-limb asymmetry and both speed and CODS performance in adult female soccer players using unilateral CMJ, DJ, and 505 testing. The findings showed significant correlations between jump-height and RSI asymmetry during the DJ test and both speed and CODS performance. This indicated that larger jumping imbalances during the DJ were associated with slower sprint and CODS times. In contrast, no significant relationships were found between CMJ asymmetry and either speed or CODS.

All tests reported good to excellent relative reliability and acceptable variability, which serves as a good indication that the data can be interpreted with confidence for further analysis [32]. The acceptable test variability is likely due to the level of experience in the present sample and indicates that structured strength and conditioning training (inclusive of speed and jump training) may have contributed to the acceptable reliability of the data. Previous data in elite female soccer players has reported CV values of 3.26% (bilateral CMJ) and 1.82%–3.30% (0–40 m sprints) [33]. The speed data in the present study is comparable (Table 1); however, the greater variability reported for jump height during both jump tests is likely attributable to the increased instability of jumping on one leg. Thus, practitioners are advised to calculate test variability in addition to outcome measures (e.g., jump height and RSI) as part of the ongoing monitoring process, not just for a single test session. Furthermore, given the lower CV values associated with bilateral jumping [33], practitioners should consider CV values <5% as a target for test variability when using unilateral jumps.

No significant relationships were present between jump-height asymmetry from the CMJ test and either speed or CODS performance (Table 2). However, significant correlations were found between DJ metrics and the 10 m, 30 m, and 505 tests. Specifically, jump-height asymmetry was correlated with the 30 m (*r* = 0.58; *p* < 0.05) and 505 (*r* = 0.52–0.66; *p* < 0.05) tests on both limbs. RSI asymmetry was correlated with the 10 m (*r* = 0.52; *p* < 0.05) and 505 (*r* = 0.54–0.55; *p* < 0.05) tests on both limbs. The relevance here is that all significant relationships were positive, indicating that larger asymmetries are associated with slower linear sprint speeds and CODS times. Without mechanistic analysis, this is challenging to fully explain. However, given the high braking properties associated with the DJ test (by virtue of falling from an elevated platform), and immediate requirement to transition into high propulsive forces straight after, it is understandable that DJ asymmetries might impact CODS performance where braking strategies are also key during a 180° turn [34,35]. This theory is in part supported by previous research from Maloney et al. [2]. It was reported that vertical stiffness and lower jump-height asymmetry from the unilateral DJ test accounted for 63% of the variance during a CODS test, although this was in recreational male adults.

In soccer, players are required to perform multiple high-intensity actions such as sprinting, changing direction, and kicking [36], which occur unilaterally and are unlikely to be performed in equal amounts using both limbs [37]. The development of asymmetry is therefore to be expected in soccer players. Nonetheless, given the associations with reduced acceleration, speed, and CODS performance in the present study, it seems plausible to suggest that targeted training strategies to reduce inter-limb asymmetry (discussed in the conclusion) may contribute to improvements in sprint and CODS performance in female soccer players.

No significant relationships were present between asymmetry scores, highlighting the independent nature of jumping and CODS asymmetry scores in adult female soccer players (Table 3). Given these findings, there is a logical conclusion to consider. Firstly, if practitioners wish to investigate asymmetry, it is likely that conducting a single test alone will not provide a complete picture of muscular imbalances among athletes. This is supported by Loturco et al. [21], who showed no significant correlations were present between asymmetries from commonly used unilateral jump tests. Furthermore, recent literature from Bishop et al. [31] showed that when comparing asymmetry scores across multiple tests, levels of agreement were typically poor. Thus, the findings in the present study are in agreement with previous research, and preclude the use of a single test to screen for inter-limb asymmetry. Given similar findings have been shown across multiple populations [21,31], it seems prudent to suggest that the lack of association between asymmetry in different tasks is less to do with female soccer players, but more to do with the variable nature of asymmetry itself.

The present study was not without some limitations. Firstly, literature has highlighted the importance of in-depth jump analyses using force plates [38,39]. Despite multiple asymmetry values for the DJ test, the CMJ only provided flight-time asymmetry scores. Thus, future research should aim to establish inter-limb differences with the use of force plates in female athlete populations. Secondly, the present study did not report findings in the context of limb dominance (i.e., kicking and non-kicking limbs) and recent research has highlighted that asymmetry may exist as a “window of opportunity” for the weaker limb [22]. It has been shown that limb dominance does not always correspond to the stronger limb in soccer players [40,41,42], nor does it in basketball or volleyball athletes [43]. Furthermore, previous research has highlighted that the weaker limb may vary according to which jump task is performed [35]. Thus, practitioners are advised to take note of the weaker limb, which may require specific attention during targeted training interventions.

## 5. Conclusions

The findings of the present study indicate that inter-limb asymmetry during unilateral DJ testing may be detrimental to speed and CODS performance; thus, practitioners may wish to consider reducing existing imbalances. From a training perspective, previous literature has suggested that reducing imbalances may be best achieved using unilateral training over bilateral methods [44,45]. A combination of strength and jumping-based exercises could be appropriate given the testing modalities employed in the present study. From a strength perspective, exercises such as split squats, single-leg squats, and step-ups will allow each limb to be better trained individually, and supplementing these with unilateral jumps in multiple directions seems like an appropriate suggestion for adult female soccer players.

## Figures and Tables

**Table 1:** Group mean scores ± standard deviations (SD), inter-limb asymmetry, and within-session reliability data for all physical performance tests.

Fitness Test	Mean ± SD	Asymmetry (%)	CV (%)	ICC (95% CI)
*UCMJ*				
Jump height-L (cm) Jump height-R (cm)	14.1 ± 2.5 13.5 ± 2.1	8.65 ± 5.98	6.47 7.51	0.90 (0.71–0.97) 0.87 (0.63–0.95)
*UDJ*				
Jump height-L (cm)	13.4 ± 2.2	9.16 ± 5.87	6.44	0.91 (0.70–0.97)
Jump height-R (cm)	12.7 ± 2.2		6.16	0.92 (0.81–0.97)
GCT-L (s)	0.29 ± 0.03	4.42 ± 3.18	3.29	0.87 (0.65–0.96)
GCT-R (s)	0.29 ± 0.03		4.58	0.88 (0.62–0.97)
RSI-L	1.13 ± 0.05	7.79 ± 5.72	3.87	0.92 (0.78–0.98)
RSI-R	1.09 ± 0.19		4.14	0.92 (0.80–0.98)
*CODS*				
505-L (s) 505-R (s)	2.55 ± 0.09 2.52 ± 0.10	2.39 ± 1.64 ^a,b^	1.01 1.31	0.94 (0.82–0.98) 0.94 (0.83–0.98)
*Speed*				
10 m (s) 30 m (s)	2.00 ± 0.10 4.89 ± 0.17	-	2.93 1.55	0.80 (0.51–0.92) 0.84 (0.55–0.94)

^a^: indicates significantly different to jump-height asymmetry in the CMJ (*p* = 0.025); ^b^: indicates significantly different to jump-height asymmetry in the DJ (*p* = 0.014); CV = coefficient of variation; ICC = intraclass correlation coefficient; CI = confidence intervals; UCMJ = unilateral countermovement jump; L = left; R = right; cm = centimetres; UDJ = unilateral drop jump; GCT = ground contact time; s = seconds; RSI = reactive strength index; CODS = change-of-direction speed; m = meters.

**Table 2:** Pearson’s *r* correlations between inter-limb asymmetry scores and both speed and change-of-direction speed scores.

Asymmetry Variable (%)	10 m	30 m	505 (left)	505 (right)
*UCMJ*				
Jump height	0.39	0.22	0.00	−0.05
*UDJ*				
Jump height	0.45	0.58 *	0.66 **	0.52 *
GCT	−0.02	−0.01	−0.21	−0.02
RSI	0.52 *	0.31	0.55 *	0.54 *
505	0.04	0.05	0.07	−0.35

**: indicates the significance of *p* < 0.01; *: indicates the significance of *p* < 0.05; m = metres; UCMJ = unilateral countermovement jump; UDJ = unilateral drop jump; GCT = ground contact time; RSI = reactive strength index.

**Table 3:** Pearson’s *r* correlations between inter-limb asymmetry scores across tests.

Asymmetry Variable	CMJ JH	DJ JH	DJ GCT	DJ RSI	505
CMJ JH	1	0.35	0.09	0.38	0.16
DJ JH	-	1	−0.27	0.45	0.39
DJ GCT	-	-	1	0.35	−0.24
DJ RSI	-	-	-	1	0.22
505	-	-	-	-	1

CMJ = countermovement jump; DJ = drop jump; JH = jump height; GCT = ground contact time; RSI = reactive strength index.

## References

[1] Jones P., Bampouras T. (2010). A comparison of isokinetic and functional methods of assessing bilateral strength imbalance. J. Strength Cond. Res..

[2] Maloney S., Richards J., Nixon D., Harvey L., Fletcher I. (2017). Do stiffness and asymmetries predict change of direction performance?. J. Sports Sci..

[3] Newton R.U., Gerber A., Nimphius S., Shim J.K., Doan B.K., Robertson M., Pearson D.R., Craig B.W., Häkkinen K., Kraemer W.J. (2006). Determination of functional strength imbalance of the lower extremities. J. Strength Cond. Res..

[4] Bishop C., Turner A., Jarvis P., Chavda S., Read P. (2017). Considerations for selecting field-based strength and power fitness tests to measure asymmetries. J. Strength Cond. Res..

[5] Sato K., Heise G. (2012). Influence of weight distribution asymmetry on the biomechanics of a barbell squat. J. Strength Cond. Res..

[6] Costa Silva J., Detanico D., Dal Pupo J., Freitas C. (2015). Bilateral asymmetry of knee and ankle isokinetic torque in soccer players u20 category. Braz. J. Kinanthrop. Hum. Perform..

[7] Ruas C., Brown L., Pinto R. (2015). Lower-extremity side-to-side strength asymmetry of professional soccer players according to playing position. Kinesiology.

[8] Coratella G., Beato M., Schena F. (2018). Correlation between quadriceps and hamstrings inter-limb strength asymmetry with change of direction and sprint in U21elite soccer players. Hum. Move. Sci..

[9] Bailey C., Sato K., Alexander R., Chiang C.-Y., Stone M. (2013). Isometric force production symmetry and jumping performance in collegiate athletes. J. Trainol..

[10] Dos’Santos T., Thomas C., Jones P., Comfort P. (2017). Assessing muscle strength asymmetry via a unilateral stance isometric mid-thigh pull. Int. J. Sports Physiol. Perform..

[11] Hart N., Nimphius S., Spiteri T., Newton R. (2014). Leg strength and lean mass symmetry influences kicking performance in Australian football. J. Sports Sci. Med..

[12] Loturco I., Pereira L., Kobal R., Cal A., Fernandes V., Ramirez-Campillo R., Suchomel T. (2018). Portable force plates: A viable and practical alternative to rapidly and accurately monitor elite sprint performance. Sports.

[13] Read P., Oliver J., De Ste Croix M., Myer G., Lloyd R. (2017). A review of field-based assessments of neuromuscular control and their utility in male youth soccer players. J. Strength Cond. Res..

[14] Kyritsis P., Bahr R., Landreau P., Miladi R., Witvrouw E. (2016). Likelihood of ACL graft rupture: Not meeting six clinical discharge criteria before return to sport is associated with a four times greater risk of rupture. Br. J. Sports Med..

[15] Bell D., Sanfilippo J., Binkley N., Heiderscheit B. (2014). Lean mass asymmetry influences force and power asymmetry during jumping in collegiate athletes. J. Strength Cond. Res..

[16] Bishop C., Read P., McCubbine J., Turner A. (2018). Vertical and horizontal asymmetries are related to slower sprinting and jump performance in elite youth female soccer players. J. Strength Cond. Res..

[17] Lockie R., Callaghan S., Berry S., Cooke E., Jordan C., Luczo T., Jeffriess M. (2014). Relationship between unilateral jumping ability and asymmetry on multidirectional speed in team-sport athletes. J. Strength Cond. Res..

[18] Dos’Santos T., Thomas C., Jones P., Comfort P. (2017). Asymmetries in single and triple hop are not detrimental to change of direction speed. J. Trainol..

[19] Maloney S., Fletcher I., Richards J. (2016). A comparison of methods to determine bilateral asymmetries in vertical leg stiffness. J. Sports Sci..

[20] Bishop C., Turner A., Read P. (2018). Effects of inter-limb asymmetries on physical and sports performance: A systematic review. J. Sports Sci..

[21] Loturco I., Pereira L., Kobal R., Abad C., Komatsu W., Cunha R., Cohen M. (2018). Functional screening tests: Interrelationships and ability to predict vertical jump performance. Int. J. Sports Med..

[22] Maloney S. (2018). The relationship between asymmetry and athletic performance: A critical review. J. Strength Cond. Res..

[23] Loturco I., Pereira L., Kobal R., Nakamura F. (2018). Using loaded and unloaded jumps to increase speed and power performance in elite young and senior soccer players. Strength Cond. J..

[24] Thomas K., French D., Hayes P.R. (2009). The effect of two plyometric training techniques on muscular power and agility in youth soccer players. J. Strength Cond. Res..

[25] Turner A., Stewart P. (2014). Strength and conditioning for soccer players. Strength Cond. J..

[26] Wisloff U., Castagna C., Helgerud J., Jones R., Hoff J. (2004). Strong correlation of maximal squat strength and with sprint performance and vertical jump height in elite soccer players. Br. J. Sports Med..

[27] Glatthorn J., Gouge S., Nussbaumer S., Stauffacher S., Impellizzeri F., Maffiuletti N. (2011). Validity and reliability of Optojump photoelectric cells for estimating vertical jump height. J. Strength Cond. Res..

[28] Koo T., Li M. (2016). A guideline of selecting and reporting intraclass correlation coefficients for reliability research. J. Chiropr. Med..

[29] Cormack S., Newton R., McGuigan M., Doyle T. (2008). Reliability of measures obtained during single and repeated countermovement jumps. Int. J. Sports Physiol. Perform..

[30] Bishop C., Read P., Lake J., Chavda S., Turner A. (2018). Inter-limb asymmetries: Understanding how to calculate differences from bilateral and unilateral tests. Strength Cond. J..

[31] Bishop C., Lake J., Loturco I., Papadopoulos K., Turner A., Read P. (2018). Inter-limb asymmetries: The need for an individual approach to data analysis. J. Strength Cond. Res..

[32] Turner A., Brazier J., Bishop C., Chavda S., Cree J., Read P. (2015). Data analysis for strength and conditioning coaches: Using excel to analyse reliability, differences, and relationships. Strength Cond. J..

[33] Haugen T., Tonnessen E., Seiler S. (2012). Speed and countermovement-jump characteristics of elite female soccer players, 1995–2010. Int. J. Sports Physiol. Perform..

[34] Graham-Smith P., Atkinson L., Barlow R., Jones P. Braking characteristics and load distribution in 180 degree turns. Proceedings of the 5th United Kingdom Strength and Conditioning Association Annual Conference.

[35] Jones P., Bampouras T., Marrin K. (2009). An investigation into the physical determinants of change of direction speed. J. Sports Med. Phys. Fit..

[36] Taylor J., Wright A., Dischiavi S., Townsend M., Marmon A. (2017). Activity demands during multi-directional team sports: A systematic review. Sports Med..

[37] Hart N., Nimphius S., Weber J., Spiteri T., Rantalainen T., Dobbin M., Newton R. (2016). Musculoskeletal asymmetry in football athletes: A product of limb function over time. Med. Sci. Sports Exerc..

[38] Bromley T., Turner A., Read P., Lake J., Maloney S., Chavda S., Bishop C. (2018). Effects of a competitive soccer match on jump performance and interlimb asymmetries in elite academy soccer players. J. Strength Cond. Res..

[39] Gathercole R., Sporer B., Stellingwerff T., Sleivert G. (2015). Alternative countermovement-jump analysis to quantify acute neuromuscular fatigue. Int. J. Sports Physiol. Perform..

[40] Rahnama N., Lees A., Bambaecichi E. (2005). A comparison of muscle strength and flexibility between the preferred and non-preferred kicking leg in English soccer players. Ergonomics.

[41] Zakas A. (2006). Bilateral isokinetic peak torque of quadriceps and hamstring muscles in professional soccer players with dominance on one or both sides. J. Sports Med. Phys. Fit..

[42] Atkins S., Bentley I., Hurst H., Sinclair J., Hesketh C. (2016). The presence of bilateral imbalances of the lower limbs in elite youth soccer players of different ages. J. Strength Cond. Res..

[43] Fort-Vanmeerhaeghe A., Gual G., Romero-Rodriguez D., Unnitha V. (2016). Lower limb neuromuscular asymmetry in volleyball and basketball players. J. Hum. Kin..

[44] Bishop C., Turner A., Read P. (2018). Training methods and considerations for practitioners to reduce inter-limb asymmetries. Strength Cond. J..

[45] Gonzalo-Skok O., Tous-Fajardo J., Suarez-Arrones L., Arjol-Serrano J.L., Casajus J.A., Mendez-Villanueva A. (2017). Single-leg power output and between-limbs imbalances in team-sport players: Unilateral versus bilateral combined resistance training. Int. J. Sports Physiol. Perform..

